# Eye tracking insights into physician behaviour with safe and unsafe explainable AI recommendations

**DOI:** 10.1038/s41746-024-01200-x

**Published:** 2024-08-02

**Authors:** Myura Nagendran, Paul Festor, Matthieu Komorowski, Anthony C. Gordon, Aldo A. Faisal

**Affiliations:** 1 UKRI Centre for Doctoral Training in AI for Healthcare, Imperial College London, London, UK; 2 Division of Anaesthetics, Pain Medicine, and Intensive Care, Imperial College London, London, UK; 3 Brain and Behaviour Lab, Imperial College London, London, UK; 4 Department of Computing, Imperial College London, London, UK; 5 Institute of Artificial & Human Intelligence, University of Bayreuth, Bayreuth, Germany

**Keywords:** Health services, Translational research, Human behaviour, Biomedical engineering, Computer science

## Abstract

We studied clinical AI-supported decision-making as an example of a high-stakes setting in which explainable AI (XAI) has been proposed as useful (by theoretically providing physicians with context for the AI suggestion and thereby helping them to reject unsafe AI recommendations). Here, we used objective neurobehavioural measures (eye-tracking) to see how physicians respond to XAI with *N* = 19 ICU physicians in a hospital’s clinical simulation suite. Prescription decisions were made both pre- and post-reveal of either a safe or unsafe AI recommendation and four different types of simultaneously presented XAI. We used overt visual attention as a marker for where physician mental attention was directed during the simulations. Unsafe AI recommendations attracted significantly greater attention than safe AI recommendations. However, there was no appreciably higher level of attention placed onto any of the four types of explanation during unsafe AI scenarios (i.e. XAI did not appear to ‘rescue’ decision-makers). Furthermore, self-reported usefulness of explanations by physicians did not correlate with the level of attention they devoted to the explanations reinforcing the notion that using self-reports alone to evaluate XAI tools misses key aspects of the interaction behaviour between human and machine.

## Introduction

Most artificial intelligence (AI) in high-stakes decision environments will be realised as part of a decision support system rather than an autonomous agent, at least in the near term^1,2^. As a result of keeping a human in the decision-making loop, evaluating the interaction and behaviour between human expert and AI is critical to improving adoption and impact at scale^3^, which has hitherto been lacking^4^. Explainable AI (XAI) is one potential way of improving this interaction between human expert and AI and is defined as an AI that, given an audience, produces details or reasons to make its functioning clear or easy to understand^5^. XAI therefore achieves its effect via an influence on human decision-making, which can be positive, negative or a mixture of both^5–7^. For example, XAI might have a positive influence by increasing general trust in an AI system though greater transparency and thereby greater accountability for end users^8^. XAI might also mitigate the potential risk of unsafe AI recommendations from being inadvertently followed by human users. The theoretical basis behind this is that humans can use the information on *why* the AI has made a suggestion to essentially ‘debug’ whether or not following the AI advice is appropriate in a given situation^9–11^. However, there are also potential negative influences on human decision-making such as automation bias (defined as the tendency for a human decision maker to disregard or not search for contradictory information in light of an AI-generated solution that is accepted as correct)^12^. Other pitfalls include the potential for the explanation itself to be incorrect, overly simplistic or be generated at the expense of trading off predictive power in a model for interpretability^13^. The available data is inconsistent on whether XAI can accomplish overall benefit (and this may well be task and user-specific in any case)^14–17^.

The medical setting is an example of a high risk decision environment with similarities to other high-stakes settings (e.g. airlines, nuclear power plants, military)^18,19^. This is in contrast to many XAI evaluations in the computer science literature focussed on low-stakes decision environments (e.g. Atari games, quizzes)^20^. Unfortunately, there are only a limited number of XAI evaluations with clinical end users (i.e. the target audience)^21^, few of which have assessed physician behaviour within a high-fidelity (i.e. more realistic or life-like) setting. In addition to this, self-reports (one of the most commonly used methods for evaluating whether XAI is actually helpful to the end-user) can be unreliable^22^. Our previous work found that self-reported XAI usefulness was a poor predictor of actual physician behaviour in a medical prescription decision task^23^. An alternative to self-reports are directly observed behavioural data that can be obtained in real-time, unlike self-reports^24^. One example is a think-aloud protocol (TAP) in which participants are asked to verbalise their decision-making process in real-time^25,26^. However, there are several limitations to TAP^27^, including the act of verbalising changing the way participants approach the task, the added cognitive load of needing to verbalise and the lack of quantitative data for analysis (usually requiring a qualitative thematic analysis instead). An alternative widely used behavioural analysis tool that also does not require reliance on self-reports and can be obtained in real-time is eye-tracking^28,29^. This technology has been used in many other non-clinical contexts for recording quantitative data on human behaviour in the form of where a participant’s attention is focused^30–34^. For eye-tracking to be effective, it ought to be employed in as high a fidelity setting as possible so that the environment more closely resembles real-world clinical practice while still allowing for experimental standardisation^35,36^. Combining eye-tracking and a high-fidelity setting therefore allows us to assess the physician-XAI interaction dynamic in a far more granular way than existing medical XAI studies.

Here, we tested four types of simultaneously presented AI explanations on clinicians in a high-fidelity simulation suite aiming to quantify how XAI influences prescription decision behaviour in a high-stakes decision environment. We therefore had two co-primary research questions. First, whether it is technically feasible to employ eye-tracking as a proxy for attention in a clinical AI task within a simulation suite. Second, whether the attention placed on XAI is greater when physicians encounter an unsafe versus safe AI suggestion (which *might* suggest that the XAI is within the causal pathway used to disregard the unsafe AI advice). Two secondary questions were (i) whether self-report data correlated with the attention proxy from eye-tracking (a lack of correlation would lend credence to self-reports being an unreliable marker for evaluating XAI benefit). And (ii) whether the attention profile (i.e. pattern of attention in a given situation or task) of physicians correlated with practice variation or propensity to follow AI advice. The reason for investigating whether attention profiles might be predictive is that if we could identify those who were more or less likely to be outliers in their prescribing behaviour or who were more or less likely to heed AI advice, it might be useful for developing more personalised AI decision-support tools (a common aim of many clinical decision support tools is to reduce unwarranted practice variation)^37^. An ability to identify outliers who rarely heed AI advice might highlight the specific users for which testing of different explanation types might be most impactful.

Our experiment was pitched at evaluating technology readiness level (TRL) 5–6 (basic validation of technology model or prototype in a relevant environment)^38^. In the experiment, physicians could encounter any of six patient scenarios, each paired with either a safe or unsafe AI recommendation (independent variable). These AI recommendations were hypothetical, designed solely to examine the interplay between physician and AI. Four kinds of explanations for the fictional AI system were created, all based on genuine types used in reinforcement learning decision support systems. For each scenario, physicians had to conduct an assessment, including reviewing patient data and examination, before being queried by a nurse about intravenous fluid and vasopressor (noradrenaline) prescriptions for the next hour of the patient’s admission. These are two commonly prescribed drugs in patients with sepsis which are used to try and improve the dysregulated circulatory profile of the patient. There can be serious (and even fatal) consequences to excessive under- and over-titration of both^39^. After viewing the AI recommendations and simultaneously presented explanations on a nearby large display, physicians were then asked to affirm or alter their prescription doses (dependent variable).

Gaze detection was employed to discern where clinicians focused their attention during the simulations. All participating physicians wore non-invasive eye-tracking glasses equipped with three cameras. One camera recorded the physician’s worldview, while the other two were trained on the physician’s eyes. Four principal regions of interest (ROIs) were outlined: the patient mannequin, the vital signs monitor, the paper ICU data chart and the AI display screen. Additional sub-regions within the AI display screen corresponded to the four types of AI explanations. ROIs were determined after post-processing the video data. Analysis included metrics such as gaze time, fixations, mean fixation duration and blink rate per ROI, all serving as indicators for attention (dependent variable). Pre- and post-experiment, participants provided self-reported data on demographics, attitudes toward AI and the utility of the AI explanations.

## Results

**Fig. 1:**
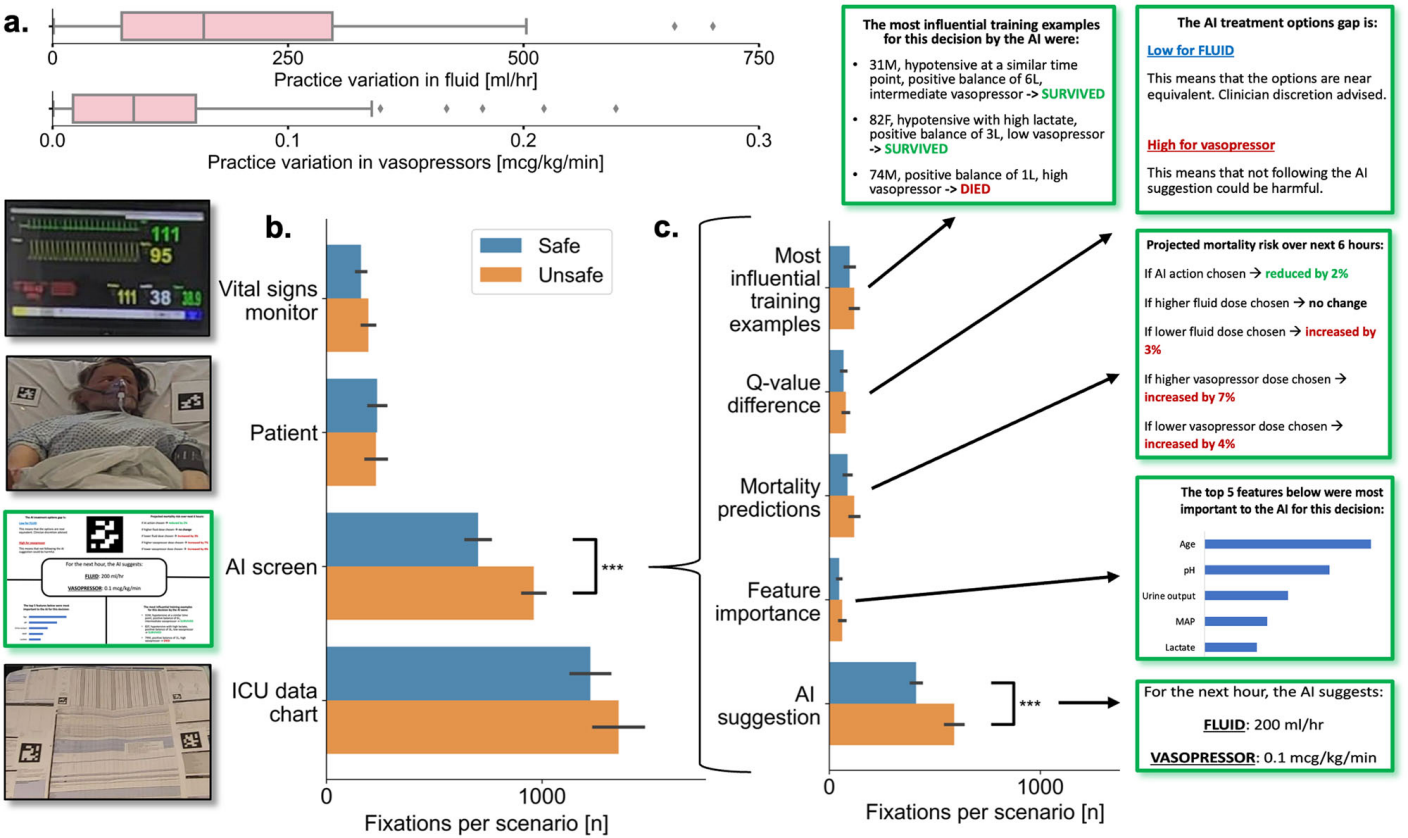
Overall practice variation for fluid and vasopressor and fixations per scenario by safety status of scenario. **a** Practice variation is defined as the absolute distance from the baseline dose chosen by the physician (i.e. pre AI-reveal) to the average dose chosen by the entire group of physicians (i.e. the metric is a proxy for the outlier extent of a given prescriber). For each boxplot, the centre line represents the median, box edges represent upper and lower quartiles, whiskers represent 1.5x inter-quartile range and diamonds are outliers. **b**, **c** Mean and SEM error bars. AI screen is a super-set encompassing the AI recommendation and all four XAI ROIs (green boxes). Significance levels are one star for *p* < 0.05 and three stars for *p* < 0.001 (based on Student’s *t*-test).

Nineteen ICU physicians with eye-tracking data available were included (13 male, 6 female). Mean physician age was 33 years (standard deviation (SD) 6 years). Mean ICU experience was 3.6 years (SD 4 years, range 2 months to 14 years). All physicians completed the task successfully with a mean scenario completion time of 5.4 min (SD 1.2 min). The mean practice variation for fluid was 217 ml/h (SD 205 ml/h) and for vasopressor was 0.04 mcg/kg/min (SD 0.04 mcg/kg/min), see Fig. 1a.

### Eye-tracking metrics on regions of interest

There were significantly more gaze fixations for the AI screen during unsafe versus safe scenarios (mean 962 [95% confidence interval (CI) 861 to 1063] vs. 704 [95% CI 593–814] respectively, *p* = 0.002 by independent T-test, Fig. 1b [alternatively displayed with box-plots in Supplementary Fig. 1]). There was no appreciable difference in the number of gaze fixations between the simultaneously presented different XAI modalities for either safe (mean 75 [95% CI 59–90]) or unsafe (mean 94 [95% CI 77–111]) scenario (Fig. 1c) as the difference for the AI screen was driven almost entirely by the AI recommendation itself rather than the XAI. However, a post-hoc power calculation suggested insufficient power to detect a significant difference with the recruited sample size. Gaze fixations stratified by both safety status and seniority of ICU physicians are shown in Supplementary Fig. 2.

**Fig. 2:**
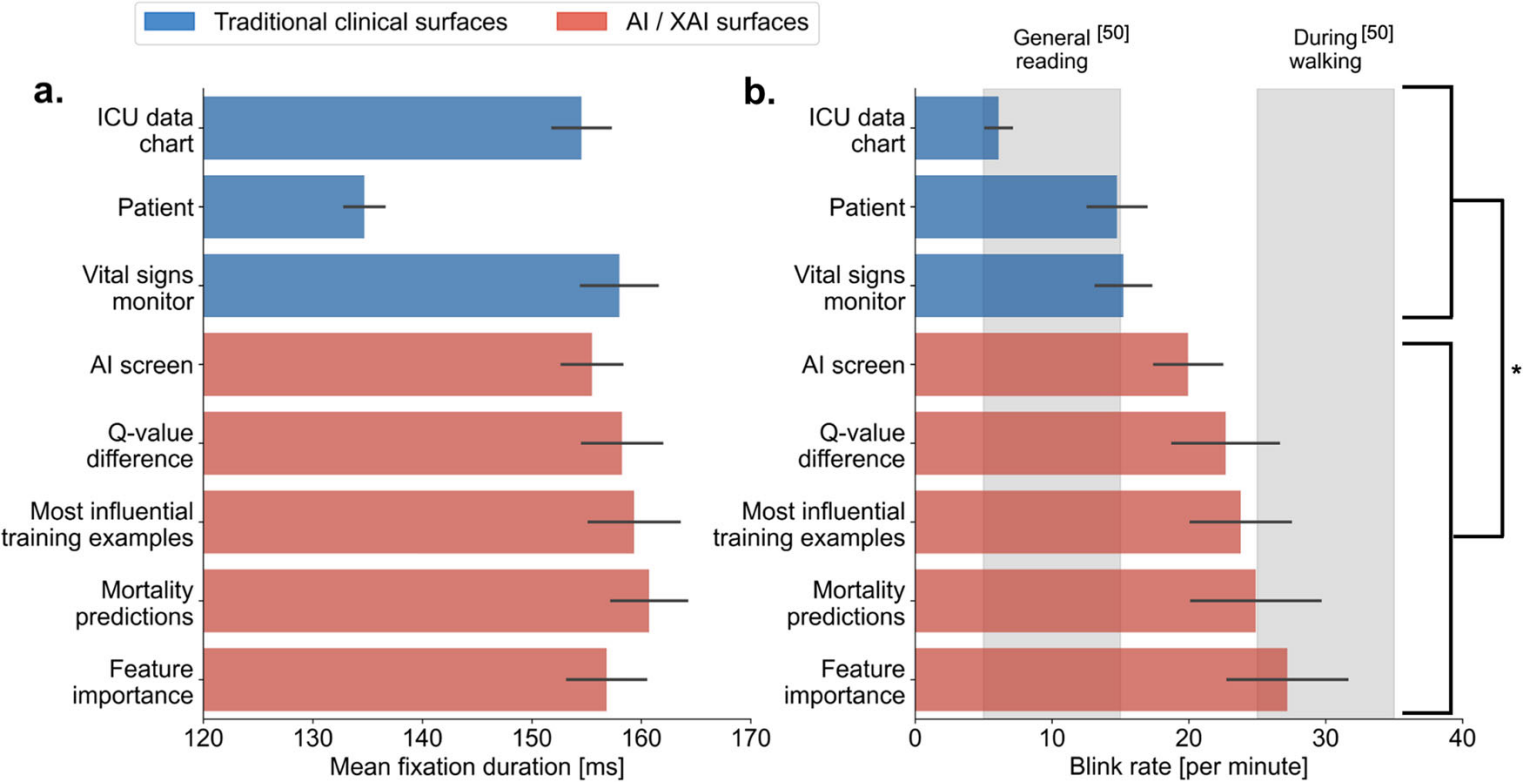
Mean fixation duration and blink rate by region of interest. Mean and SEM error bars. Blue bars are traditional clinical surfaces while red bars are AI / XAI surfaces. The left hand (**a**) shows fixation duration while the right hand (**b**) shows blink rate. † The grey shaded regions show typical blink rate estimates associated with either general reading (requiring more concentration) and walking (less concentration) as per Chidi-Egboka et al. Invest Ophthalmol Vis Sci. 2023 [ref. ^50^ in manuscript]. Significance levels are one star for *p* < 0.05 and three stars for *p* < 0.001 (based on Student’s *t* test).

Mean fixation duration was lowest for the patient mannequin (135 ms, SD 8 ms) and similar for all other surfaces (see Fig. 2a [alternatively displayed with box-plots in Supplementary Fig. 3]). Mean blink rate was lowest for the ICU chart (6.1 blinks per minute (bpm), SD 4.1), similar for both vital signs monitor and patient mannequin (mean 15.2 bpm and 14.7 bpm, SD 8.7 and 9.2 respectively) and notably higher for the AI screen (mean 19.9 bpm, SD 10.7), see Fig. 2b [alternatively displayed with box-plots in Supplementary Fig. 3]. When comparing all conventional clinical ROIs (chart, patient mannequin, monitor; blue bars in Fig. 2b) to all AI ROIs (including XAIs; red bars in Fig. 2b), there was a significantly lower mean blink rate on the conventional clinical ROIs than the AI ROIs (12.0 bpm vs. 23.7 bpm, *p* = 0.002 by independent T-test).

We also assessed gaze per ROI in a manner that took into account ‘visual real-estate’ (i.e. the proportion of the participant’s worldview occupied by the ROI) by comparing the actual gaze proportion to that expected by chance alone. This allowed us to compare across ROIs (despite them being different sizes and clinicians moving across the room). For every ROI except the patient mannequin, there was a significantly higher actual than chance gaze proportion (*p* < 0.001 for all comparisons except patient mannequin, all by independent T-test, see Supplementary Fig. 4). For the major ROIs (AI screen, ICU chart, vital signs monitor, patient mannequin) the ratio of actual to chance gaze was 6.5, 1.6, 12.5 and 1.3 respectively. For the XAI ROIs (training examples, *Q* value difference, mortality, feature importance) the ratio of actual to random gaze was 6.1, 4.2, 5.3 and 3.3 respectively (see Supplementary Fig. 5).

### Clinical practice variation among physicians

**Fig. 3:**
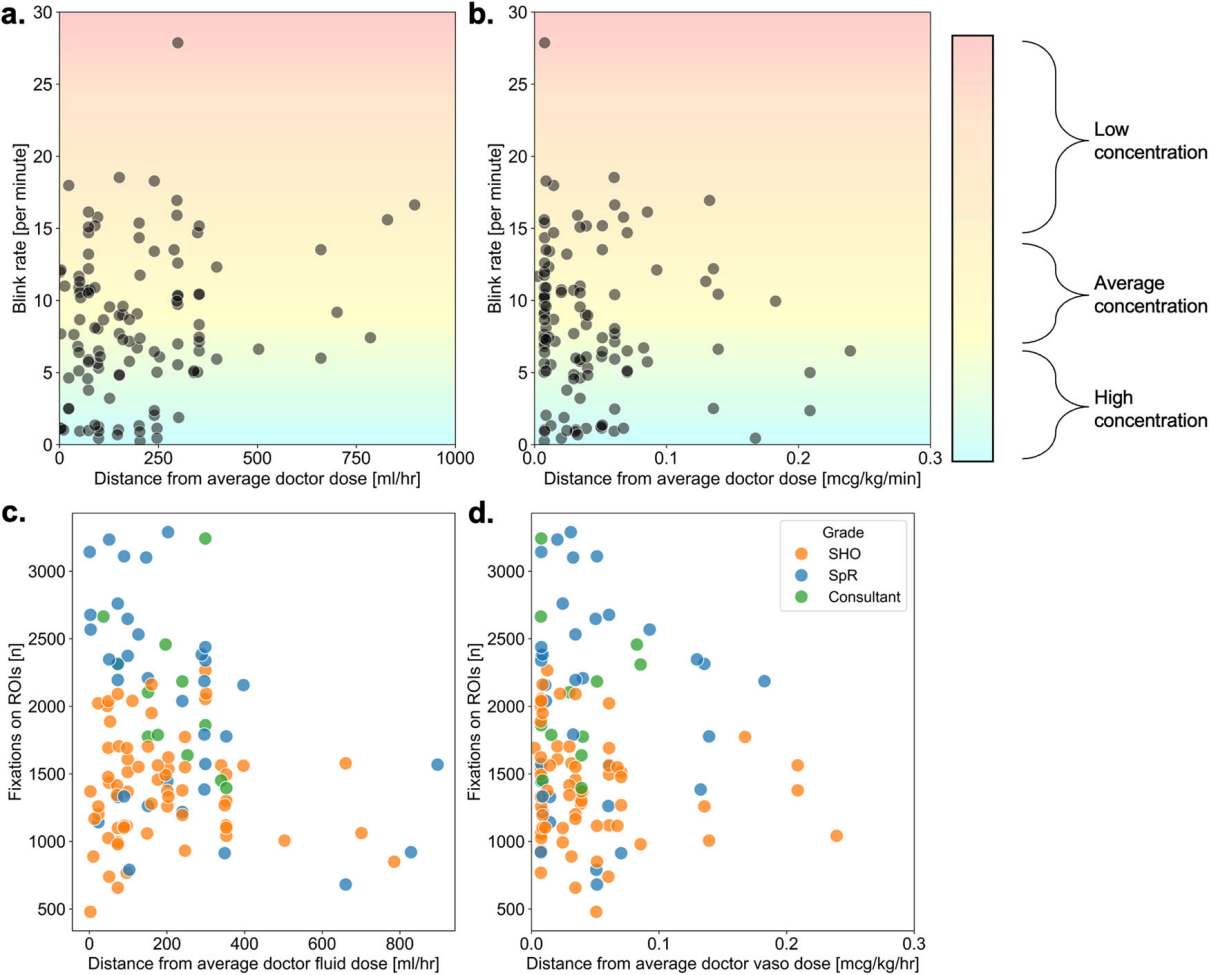
Correlation between practice variation and eye tracking metrics, for both fluid and vasopressor. **a** Blink rate versus practice variation for fluid, (**b**) blink rate versus practice variation for vasopressor, (**c**) number of gaze fixations versus practice variation for fluid, (**d**) number of gaze fixations versus practice variation for vasopressor. The degree of practice variation is defined by the per trial distance between a physician’s prescription and the average prescription dose of all physicians. The background shading of plots (**a**) and (**b**) gives an indicator for expected blink rate depending on level of focus/concentration. Typical spontaneous blink rate is around 8–15 with lower levels suggesting higher focus/concentration and vice versa.

We defined practice variation as the dose distance of an individual physician from the average of all pre-AI reveal physician prescriptions in any given trial/scenario (higher dose distance suggesting that the physician was more of an outlier in that particular trial and vice versa). Overall, there was no strong pattern between eye-tracking metrics (number of gaze fixations and blink rate) and the degree of clinical practice variation (see Fig. 3).

### Influence of AI and simultaneously provided XAI on physicians

**Fig. 4:**
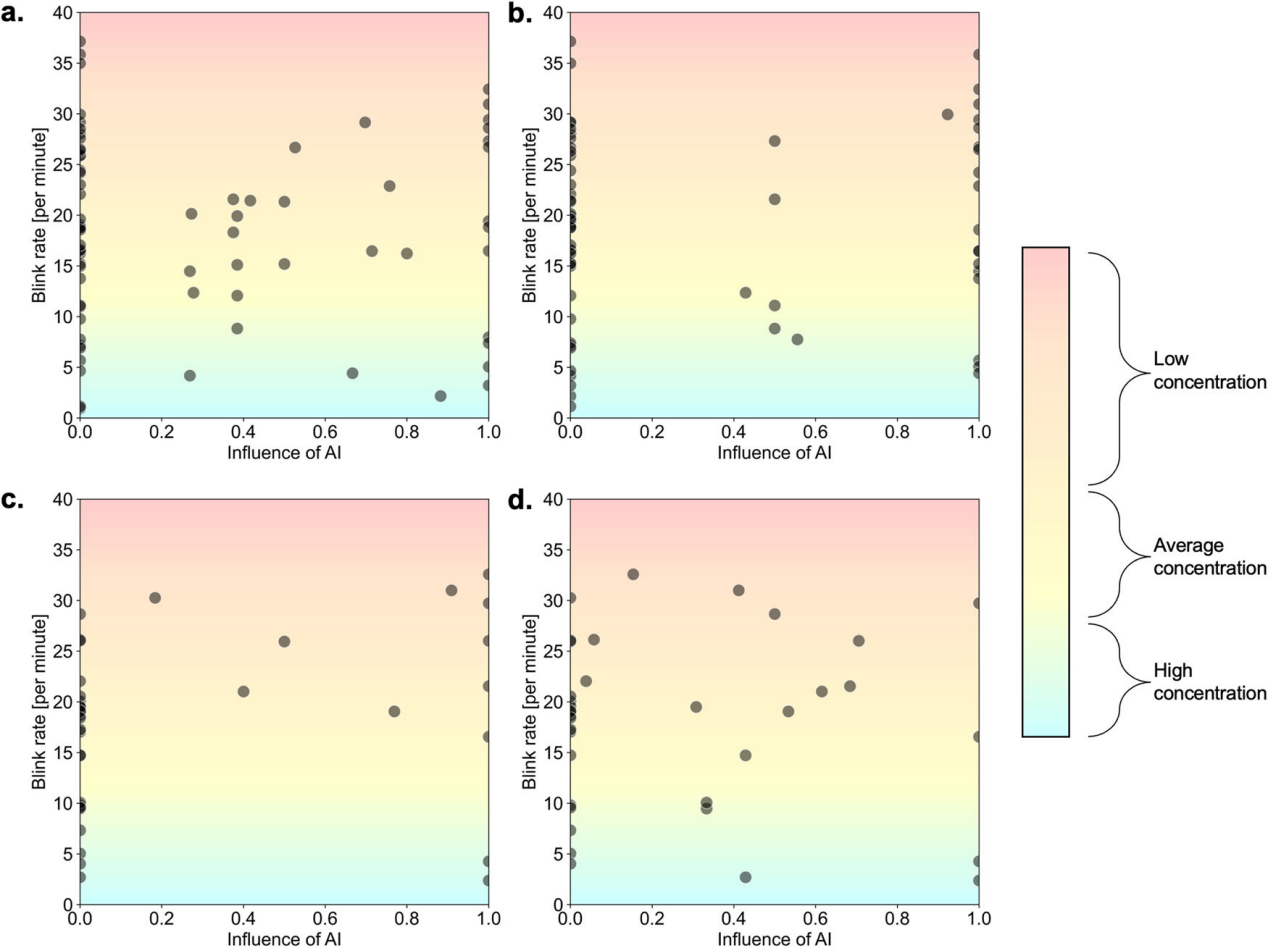
Blink rate by influence of AI for both fluid and vasopressor and for both safe and unsafe AI suggestions. **a** Blink rate versus influence of AI for fluid with safe AI recommendation, (**b**) blink rate versus influence of AI for vasopressor with safe AI recommendation, (**c**) blink rate versus influence of AI for fluid with unsafe AI recommendation, (**d**) blink rate versus influence of AI for vasopressor with unsafe AI recommendation. Influence of AI was calculated on a continuous scale from 0 (completely ignoring advice) to 1 (completely relying on advice) using the formula [(final estimate − initial estimate)/(advice – initial estimate)] per work by Yaniv et al. The background shading of the plots gives an indicator for expected blink rate depending on level of focus/concentration. Typical spontaneous blink rate is around 8–15 with lower levels suggesting higher focus/concentration and vice versa.

**Fig. 5:**
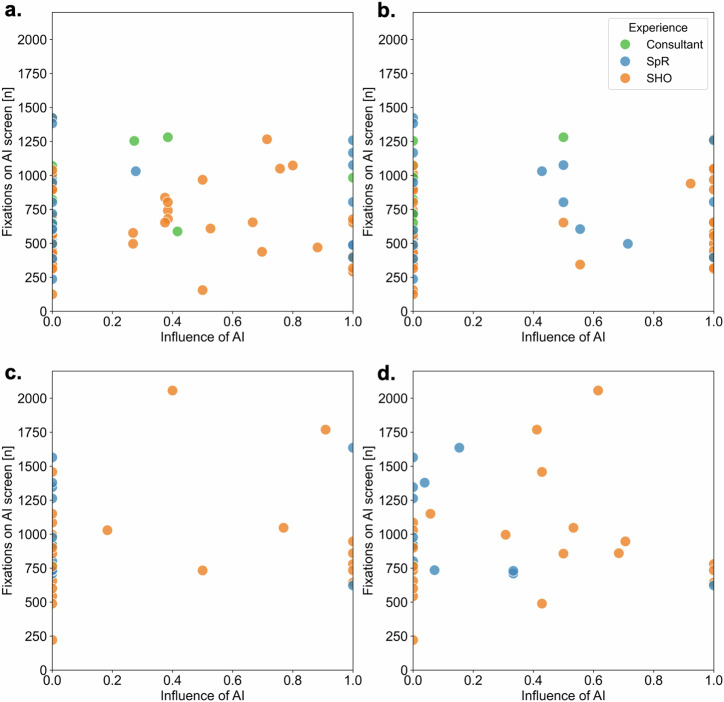
Number of gaze fixations by influence of AI for both fluid and vasopressor and for both safe and unsafe AI suggestions. **a** Gaze fixations versus influence of AI for fluid with safe AI recommendation, (**b**) gaze fixations versus influence of AI for vasopressor with safe AI recommendation, (**c**) gaze fixations versus influence of AI for fluid with unsafe AI recommendation, (**d**) gaze fixations versus influence of AI for vasopressor with unsafe AI recommendation. Influence of AI was calculated on a continuous scale from 0 (completely ignoring advice) to 1 (completely relying on advice) using the formula [(final estimate − initial estimate)/(advice − initial estimate)] per work by Yaniv et al. Points are also categorised by grade of experience (Consultant, most senior and equivalent to attending in the United States (US); SpR, specialist registrar and equivalent to fellow in the US; SHO, senior house officer and equivalent to resident in the US).

We found no strong evidence of correlation between blink rate and influence of AI regardless of safety status or drug (see Fig. 4). Nor was there strong evidence of correlation between number of gaze fixations and influence of AI, again regardless of safety status or drug (see Fig. 5). There was no change when instead looking at the distance between a physician’s final prescription (having had the opportunity to view the AI recommendation) and the value of the AI recommendation rather than influence of AI per se (see Supplementary Figs. 6 and 7). The absolute distance between final prescription and AI recommendation for fluid was 171 ml/h and 155 ml/h respectively for safe and unsafe conditions. For vasopressor, the distance between a physician’s final prescription and the value of the AI recommendation was 0.04 mcg/kg/min and 0.30 mcg/kg/min respectively for safe and unsafe conditions.

### Self-reported XAI usefulness

The overall mean post-experiment usefulness rating for the XAI was 3.2 (SD 1.0) on a 0–4 scale with higher value implying the XAI was more useful. The training examples explanation was the only one of the four to be rated significantly lower than the overall rating for explanations in general (mean 1.4 versus 3.2, SD 1.3 versus 1.0, *p* < 0.001 by T-test, see Supplementary Fig. 8). When comparing the ‘objective’ marker of how many fixations there were on the four different types of XAI to the ‘subjective’ marker of how clinicians rated the usefulness of the four XAIs, we found no correlation for any XAI (see Supplementary Fig. 9).

## Discussion

This study has several important findings that add to our understanding of physician behaviour during their interaction with AI-driven decision support tools and the accompanying explanations. First, measuring gaze fixations and blink rate as a proxy for attention to an AI support tool was feasible within a high-fidelity simulation environment. Whether or not this could be extended to real-world clinical settings would depend on availability of less-intrusive eye-tracking hardware and appropriately addressing privacy concerns arising from video recordings of staff and patients. Second, while unsafe AI recommendations attracted greater attention than safe AI recommendations, there was not clearly higher attention placed onto any of the four types of simultaneously presented explanation during an unsafe recommendation (i.e. there was no evidence of extra reliance on explanations during the unsafe scenarios). However, we lacked power to detect a significant difference in the XAI condition. Third, self-reported usefulness of explanations by physicians did not correlate with the level of attention they devoted to the explanations. This reinforces the notion that using self-reports alone to evaluate XAI tools misses key aspects of the interaction behaviour between human and machine. Fourth, we were unable to find strong patterns between eye-tracking metrics and either clinical practice variation among physicians or their influenceability by AI recommendations.

**Fig. 6:**
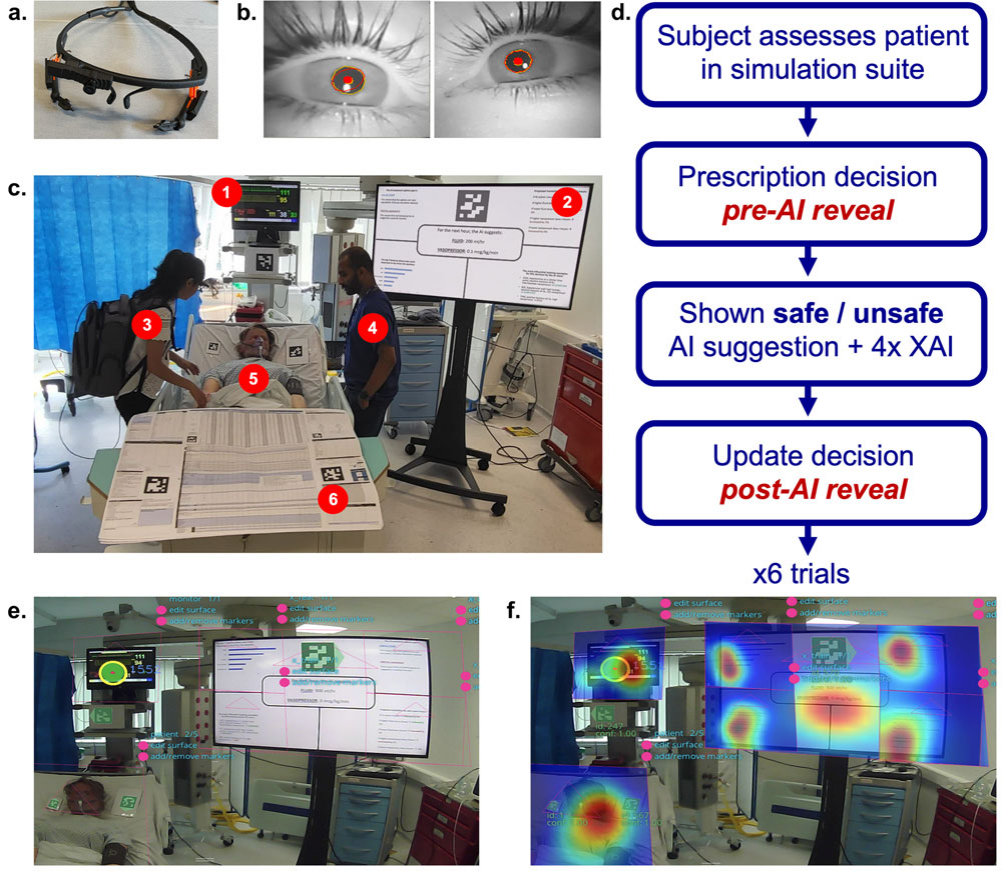
Eye-tracking setup within the simulation suite. **a**, **b** Eye-tracking glasses and pupil detection. In (**a**), the eye-tracking glasses contain three cameras. One front-facing camera captures the participant world-view (i.e. Figure **e** view) while two cameras (one in front of each eye) record pupil movement and position, as visualised by the red dot and circles in (**b**). **c** Simulation suite. (1) vital signs monitor, (2) AI screen, (3) physician, (4) bedside nurse (played by experimenter), (5) high-fidelity patient mannequin, (6) bedside ICU data chart. **d** Experiment protocol. All physicians encountered six patient scenarios (four with safe AI and two with unsafe AI). **e**, **f** Video post-processing. In 6e, QR code tags (highlighted in green) are auto detected and allow regions of interest (ROIs) to have manual bounding boxes drawn. In (**f**), heatmaps are shown for gaze proportion within each of the annotated ROIs.

These findings should be considered in the context of several major limitations. First, while the fidelity of the simulation suite is far closer to real-world practice than a vignette experiment, it nonetheless misses key features of the real hospital environment. For example, the ability to dynamically examine a patient, observe them over time (rather than a snapshot scenario) and to interact with multiple other colleagues within a multi-disciplinary team before considering an AI recommendation. Unfortunately, these same features also make it near impossible to standardise an experiment in a real hospital and thus require prohibitively large sample sizes. Therefore simulation experiments still have a critical role in the initial exploration of human-AI interaction dynamics before larger scale real-world studies. Second, the explanations were presented simultaneously (Fig. 6c). We therefore cannot disentangle the marginal contribution of each had they been seen in isolation (to do this would have required a factorial trial design with at least 10 arms) and instead can only comment on the overall impact of seeing all four explanations together. Third, our sample was small and we therefore cannot exclude the possibility that some of the comparisons might have been significantly different if we had been able to include more physicians (principally more senior/experienced physicians), especially the XAI comparisons. The variable performance of the eye-tracking glasses and software in consistently detecting pupils was low among some excluded physicians which further reduced the sample available for analysis. Given the sample size, we cannot say for certain whether non-significant findings are due to lack of a true effect or too few participants.

Fourth, the demarcation between what constitutes a safe or unsafe AI recommendation necessarily imposes an arbitrary boundary on a continuous spectrum (for which no ‘gold standard’ answers exist). We used deliberately extreme unsafe scenarios in this study to allow us to capture lower bounds on the propensity to inadvertently follow dangerous AI advice. Therefore our findings might be more extreme for a set of borderline AI recommendations which many clinicians might feel uncomfortable about following but that would not have fulfilled our definition of ‘unsafe’. Fifth, some explanations were predominantly graphical (feature importance) and others were more text-heavy (influential training examples). It stands to reason that less gaze would be required to parse the former than the latter and this might have confounded the comparisons between explanations. Another potential confounder which we did mitigate against was the location of each type of explanation (as there might be a bias toward, for example, the top left of the screen). This was mitigated against by rotating the position of explanations between trials (i.e. the XAI based on feature importance would not always be in the same corner of the screen). Sixth, the explanations for both safe and unsafe conditions in the same patient scenario were identical. This inadvertently introduces another experimental condition (explanation quality) which in some studies has been the exclusive focus of the experiment^13^. An experiment with 6 arms might have better assessed this condition (two arms: safe/unsafe AI against 3 arms: good quality/poor quality/absent XAI = 6 arms). Seventh, the large TV screen is not typical of the display used for most hospital electronic health record (EHR) systems (EHR is typically displayed via laptops or desktop monitors). It was chosen to maximise discrimination of gaze between the explanation types in each corner of the screen. Future experiments should not only look to use smaller screens but also to embed the XAI being evaluated into a dummy version of the real hospital EHR software for maximum fidelity to real-world practice.

Despite these limitations, reviewing our findings alongside existing literature provides critical insights into how we might improve the deployment of XAI-based medical decision support tools. The behavioural intuition that explanations ought to help users to correctly reject poor or unsafe AI advice typically goes as follows: (i) the user is presented with an unsafe AI recommendation, (ii) the user is then provided an explanation for the AI recommendation, (iii) the user either notices a deficiency in the explanation or fails to find mitigating circumstances that explain why the AI recommendation is inappropriate, (iv) the user rejects the unsafe AI advice. One study in non-clinicians provides evidence that the causal link between (iii) and (iv) in the chain might be absent^40^. These authors found that the influence of AI advice was greater when provided alongside an explanation but that the quality of explanation did not seem to make a difference, i.e. participants may have fallen into an automation bias trap by using explanation presence alone as a heuristic for AI advice worthy of being followed (rather than actually evaluating the content of the explanation). This danger of automation bias has been highlighted in other clinical studies too^41,42^. Further evidence includes an experiment assessing a mental health drug decision support tool. Explanations did not rescue clinical users from following intentionally poor AI recommendations^15^. In our study, we confirmed that the significantly higher rejection rate of unsafe compared to safe advice could not be clearly explained by a greater reliance on, or attention devoted to, simultaneously presented explanations. Unlike previous experiments, we confirmed this by assessing the trifecta of what physicians did (i.e. their actual prescription decisions), how they did it (i.e. what they looked at during the decision-making process) and what they said (i.e. their subjective ratings for the explanations after the experiment ended).

We did not specifically assess algorithmic aversion in this study (the opposite phenomenon to automation bias where there is instead inappropriate reluctance to use AI) because in a medical setting, this would tend towards the current standard of care (status quo) and therefore be less dangerous, at least from a medico-legal standpoint^43^. However, the behavioural mechanisms for algorithmic aversion have been elucidated by others and seem related to the subjective difficulty of understanding AI and the presumed illusory subjective understanding of human decision-making itself^44^. It seems likely that progress in clinical XAI will need to address both automation bias and algorithmic aversion. As a result, being able to identify where a physician falls on this spectrum and being able to individualise the XAI output will be important. We assessed whether eye-tracking of physician behaviour might achieve this.

While there has been little use of eye-tracking technology in AI-user studies to date, one notable example includes work by Cao and colleagues who used a spatial reasoning task and found positive correlation between percentage gaze on the AI recommendation and both perceived user reliance on AI as well as their agreement with AI recommendations^24^. However, gaze did not seem to correlate with perceived trust. Though we did not measure perceived trust per se, we also found no correlation between subjective explanation rating and number of gaze fixations on the AI explanations. One proposed advantage of using eye-tracking to evaluate human-AI interactions is that it occurs in real-time (unlike post-hoc subjective ratings or human agreement with AI recommendations) and therefore could form the core of an adaptive collaboration feedback loop^24^. Our findings suggest that before eye-tracking can become a central aspect of such a feedback system incorporating XAI, we first need to establish more robust phenotypic patterns of eye movements that accurately categorise users and ideally even predict their behavioural interactions with an AI system.

Taken together, our early and exploratory findings have important implications for the design and evaluation of real-world XAI systems. We used the exemplar of a complex high-stakes decision-making process (in this case acute healthcare) to show that it is feasible to perform eye-tracking to evaluate human behaviour with simultaneously presented XAI and that the response to safe or unsafe AI recommendations is identifiably different. Notwithstanding the study limitations, the lack of clear rescue provided by simultaneously presented XAI calls into question its utility as a mitigation against harm from experts (in our case physicians) erroneously following poor quality AI advice. AI in healthcare contains all of the elements that make real-world AI deployment a hard problem. We believe that a more empirical focus on quantitative behavioural studies with human expert end-users, as we conducted here and in our accompanying manuscript on safety^45^, will become increasingly important not only for regulatory approval^46–48^ but also for cultivating physician trust and acceptance^8^.

## Methods

### Experiment conditions and AI decision support system

We performed an observational human-AI interaction study in a simulation suite. Each of six patient scenarios could be encountered by a physician under one of two conditions (see trial matrix in Supplementary Note 1): safe (four cases) or unsafe (two cases) AI recommendation. The categorisation of recommendations to safe or unsafe was based on extreme over or underdosing of fluid and vasopressor as per previous work^49^. The AI recommendations themselves were synthetic as the purpose of this experiment was to test interaction dynamics between physician and AI. Four types of explanations for the fictitious AI system were constructed (all based on realistic explanation types that we have applied to reinforcement learning decision support systems). Offline reinforcement learning is a variant of reinforcement learning where an agent learns from a fixed dataset of previous interactions (consisting of states, actions and rewards) without real-time environmental feedback. This approach aims at maximising cumulative rewards based on historical data, enabling decision-making in situations where direct interaction is impractical or risky. Our four explanation types included the following: first, a natural language description of the Q-value difference between the recommended action and alternative actions (a marker of the extent to which the optimal AI recommendation is significantly better than the alternative or only marginally better). Second, the change in short-term mortality after dosing changes as predicted by the AI. Third, the top-five ranked feature importance for input data contributing to the AI recommendation. Fourth, the three most influential training examples during the Q-learning process. Further details are given in Supplementary Note 2.

The explanations for both safe and unsafe conditions in the same patient scenario were identical. Varying the explanations between safe and unsafe conditions would introduce another factor (explanation quality) which would have required more arms to the experiment (e.g. 12 arms in the experiment by Shafti and colleagues; AI performance good/bad (2) × AI explanation good/poor/none (3) × explanation in training (2) = 2 × 3 × 2 = 12 arms)^40^. We therefore felt the simpler and more intuitive choice for this early exploratory study was to not vary the explanation between safe and unsafe scenarios but this should be acknowledged as a limitation.

### Gaze detection via eye-tracking

We used gaze detection as a proxy for where clinician attention was directed during the simulations and how this varied. All physicians wore non-invasive commercially available eye-tracking glasses (Pupil Core headset and Pupil Labs software, Core, version 3.3) with three cameras in total (Fig. 6a). The first was a camera that recorded the world-view from the physician’s perspective. The others were a pair of cameras focused on the physician’s eyes. The Pupil Labs software (Pupil Capture, version 3.5.7) used both eye cameras to demarcate the pupil and calculate where in the world-view gaze was directed (Fig. 6b).

A pre-experiment 2D calibration exercise was performed consisting of two parts. The first was a static calibration exercise using five screen markers on the laptop screen (default Pupil Labs ‘screen marker’ calibration). This was followed by a depth-based static exercise with physicians sequentially focusing on nine screen markers (‘natural features’ mode) on a 60-in. TV screen, initially at 1 m and then 2 m distance from the screen. The change in depth assisted with calibration to a real-world environment where participants were able to move their head naturally. The eye-tracking glasses were connected to a laptop (Lenovo Thinkpad) for the duration of the experiment. The laptop was placed into a lightweight rucksack worn by participants and battery powered so as to allow physicians free movement in the suite.

Four key regions of interest (ROIs) were defined (Fig. 6c): the patient mannequin (Simman 3G, Laerdal Medical, Stavanger, Norway), the vital signs monitor, the paper intensive care unit (ICU) data chart and the AI display screen. Within the last of these, four further sub-regions were identified corresponding to the four types of explanation for the AI recommendation. ROIs were identified in post-processing via identification of pre-placed QR codes (known as April tags, see Fig. 6e) within the simulation suite that could be used to define ROI boundary boxes. The following eye-tracking metrics could be analysed after post-processing: (i) gaze-time per ROI, (ii) fixations per ROI (a fixation is the most common eye-movement and occurs when eyes cease scanning and hold the foveal area of the visual field in a single place), (iii) mean fixation duration per ROI and (iv) blink rate (per minute) per ROI. Blink rate is usually inversely correlated to concentration or focus on an object and previous literature suggests that blink rate is not affected by ambient brightness unlike pupil diameter^50^. All four metrics provide a proxy for attention^50–55^.

### Simulation experiment

Physicians initially completed a standardised experiment briefing (see Supplementary Note 3) as well as a pre-experiment questionnaire on demographics and attitudes toward AI (see Supplementary Note 4). They were then taken into the simulation suite and oriented before conducting the eye-tracking calibration exercises. An experimenter played the role of the bedside ICU nurse. The physician was tasked with assessing six simulated ICU patients with sepsis as per Fig. 6d (see Supplementary Note 5 for further details on scenarios). Prior to the experiment, the six simulated cases (designed by the three doctors in the investigator team) were piloted on doctors from the Imperial College London Critical Care Research Group. Results of the piloting showed 93% agreement with safe/unsafe ratings (see Supplementary Note 6 for further details). The rationale behind selecting the six cases was to use typical sepsis scenarios that are common among the UK ICU clinical experience (pneumonia, urosepsis, COVID, intra-abdominal sepsis secondary to GI perforation, endocarditis, necrotising fasciitis).

Within each of the six scenarios, they were tasked with conducting an assessment (to include review of the available patient data and patient mannequin examination) before being asked by the bedside nurse for a fluid and vasopressor prescription for the next hour of the patient’s admission. Physicians were then shown the AI recommendation and four simultaneously presented explanations on a large display adjacent to the patient bed. They were subsequently asked to confirm or change their prescription doses (see Fig. 6d). We mitigated for the fact that unsafe AI suggestions might impact the confidence of a doctor for future scenarios by (i) ensuring that the first scenario any doctor encountered was always a safe scenario and (ii) using a trial matrix that meant different doctors encountered the unsafe scenarios in different orders (see Supplementary Note 1).

Influence of AI was calculated on a continuous scale from 0 (completely ignoring advice) to 1 (completely relying on advice) using the formula [(final estimate − initial estimate)/(advice − initial estimate)] per work by Yaniv et al. ^56^. We also repeated our analysis using a different metric: the distance between a physician’s final prescription (having had the opportunity to view the AI recommendation) and the value of the AI recommendation for any given trial/scenario (higher distance suggesting that the physician might have been *less* adherent to AI and vice versa).

### Participant recruitment and experiment conduct

ICU physicians were recruited as participants using both targeted advertising to a local NHS trust (Imperial College Healthcare NHS Trust) and convenience sampling with the following inclusion criteria: (i) practising physician, (ii) has worked for at least 2 months in an adult ICU, (iii) currently works in ICU or has worked in ICU within the last 6 months. Each experiment lasted approximately 60 minutes in total and participants were compensated for their time. All participants provided informed consent to participate. The study was approved by the Research Governance and Integrity Team (RGIT) at Imperial College London and the Health Research Authority (Ref 22:/HRA/1610). Images of participants and experimenters in Fig. 6 were obtained with written informed consent (there are no patients in this study).

### Reporting summary

Further information on research design is available in the Nature Research Reporting Summary linked to this article.

### Supplementary information

Supplementary material

Reporting Summary

## Data Availability

The data (in CSV format) that support the findings of this study are available online at: 10.6084/m9.figshare.23192615.

## Code availability

The code (in the form of Jupyter notebooks) that support the findings of this study are available online at: 10.6084/m9.figshare.23192615.
