# Supplementary material for: Eye tracking insights into physician behaviour with safe and unsafe explainable AI recommendations
Source: NPJ Digit Med. 2024 Aug 2;7:202. doi: 10.1038/s41746-024-01200-x (PMC11297294; doi:10.1038/s41746-024-01200-x)
Supplement: Supplementary file 1 — Supplementary material [file 41746_2024_1200_MOESM1_ESM.pdf]

SUPPLEMENTARY Figure 1

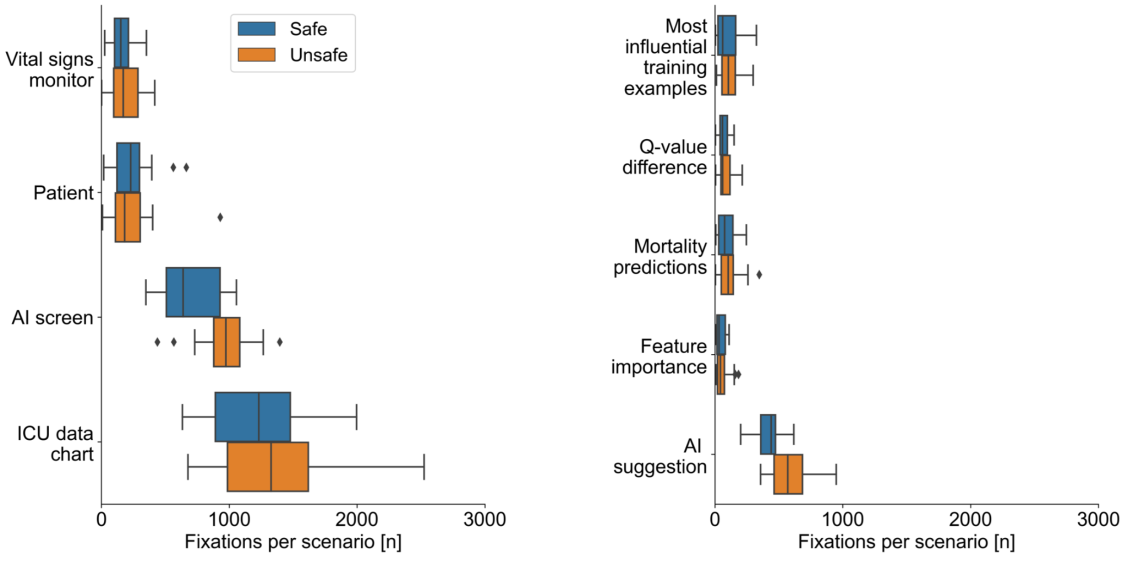

Fixations per scenario by safety status of scenario. Analogous to Figure 1b and 1c in the manuscript but box-plots rather than bar plots. AI screen is a super-set encompassing the AI suggestion and all four XAI ROIs. For each boxplot, the centre line represents the median, box edges represent upper and lower quartiles, whiskers represent 1.5x inter-quartile range and diamonds are outliers.

SUPPLEMENTARY Figure 2

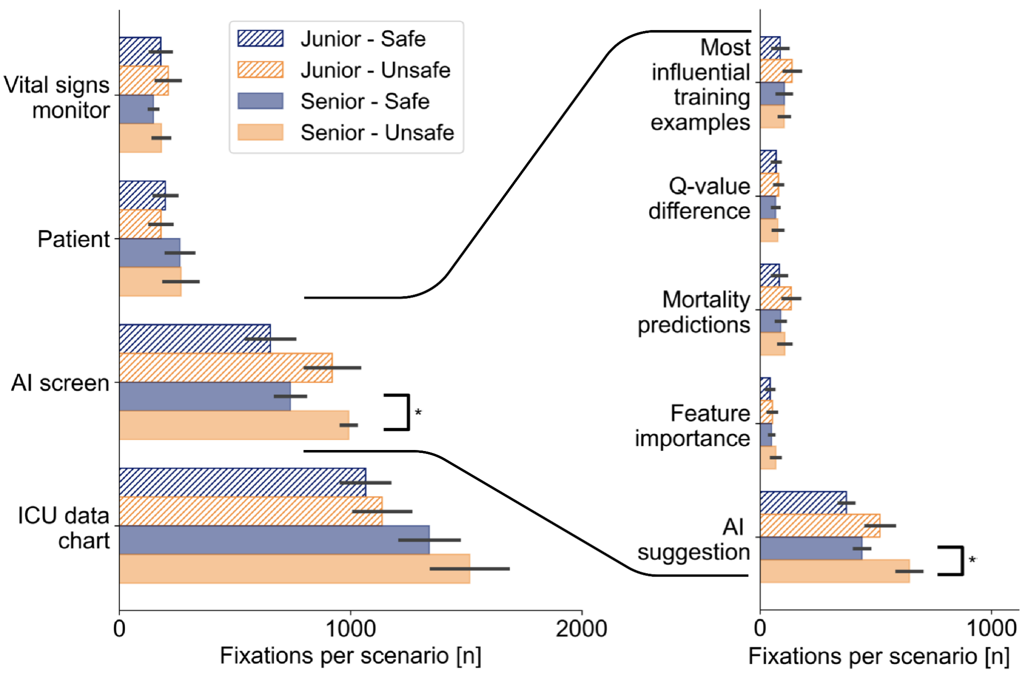

Fixations per scenario by safety status of scenario and experience level of doctor. Mean and SEM error bars. The AI screen is a super-set encompassing the AI suggestion and all four XAI ROIs. Significance levels are one star for $p<0.05$ and three stars for $p<0.001$ (based on Student's t-test).

SUPPLEMENTARY Figure 3

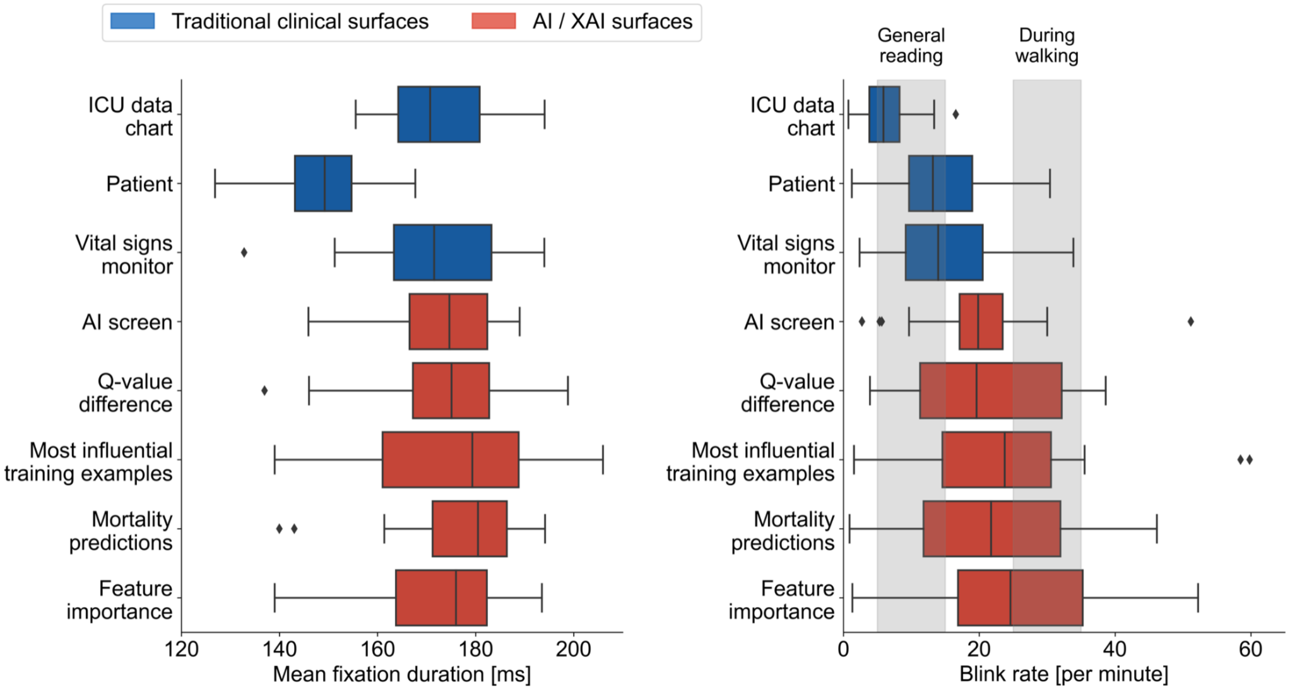

Mean fixation duration and blink rate by region of interest. Analogous to Figure 2 in the manuscript but box-plots rather than bar plots. For each boxplot, the centre line represents the median, box edges represent upper and lower quartiles, whiskers represent 1.5x inter-quartile range and diamonds are outliers. Blue bars are traditional clinical surfaces while red bars are AI / XAI surfaces. The left hand figure shows fixation duration while the right hand figure shows blink rate. The grey shaded regions show typical blink rate estimates associated with either general reading (requiring more concentration) and walking (less concentration) as per Chidi-Egboka et al. Invest Ophthalmol Vis Sci. 2023 [reference 50 in the manuscript].

SUPPLEMENTARY Figure 4

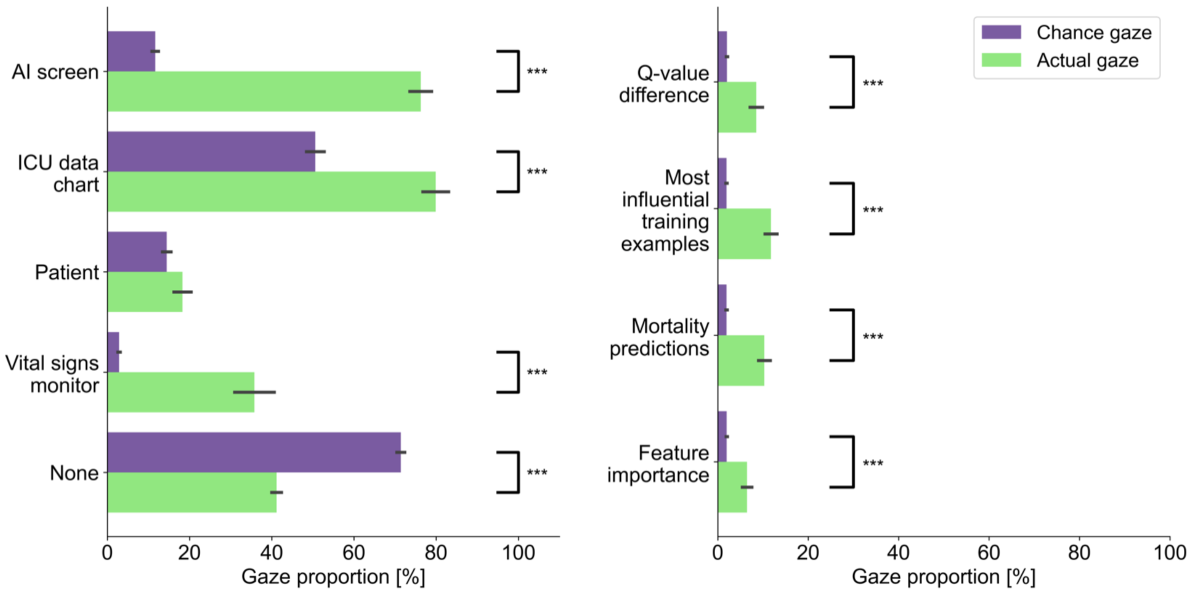

Gaze proportion per ROI. Mean and SEM error bars. Chance gaze is the proportion expected based on the area occupied by an ROI within the visual field (i.e. if ROI takes up 50% of the screen, 50% of the time, we would expect gaze to fall within it 25% of the time by chance alone). Actual gaze is the proportion observed during the experiment. Significance levels are one star for p<0.05 and three stars for p<0.001 (based on Student's t-test).

SUPPLEMENTARY Figure 5

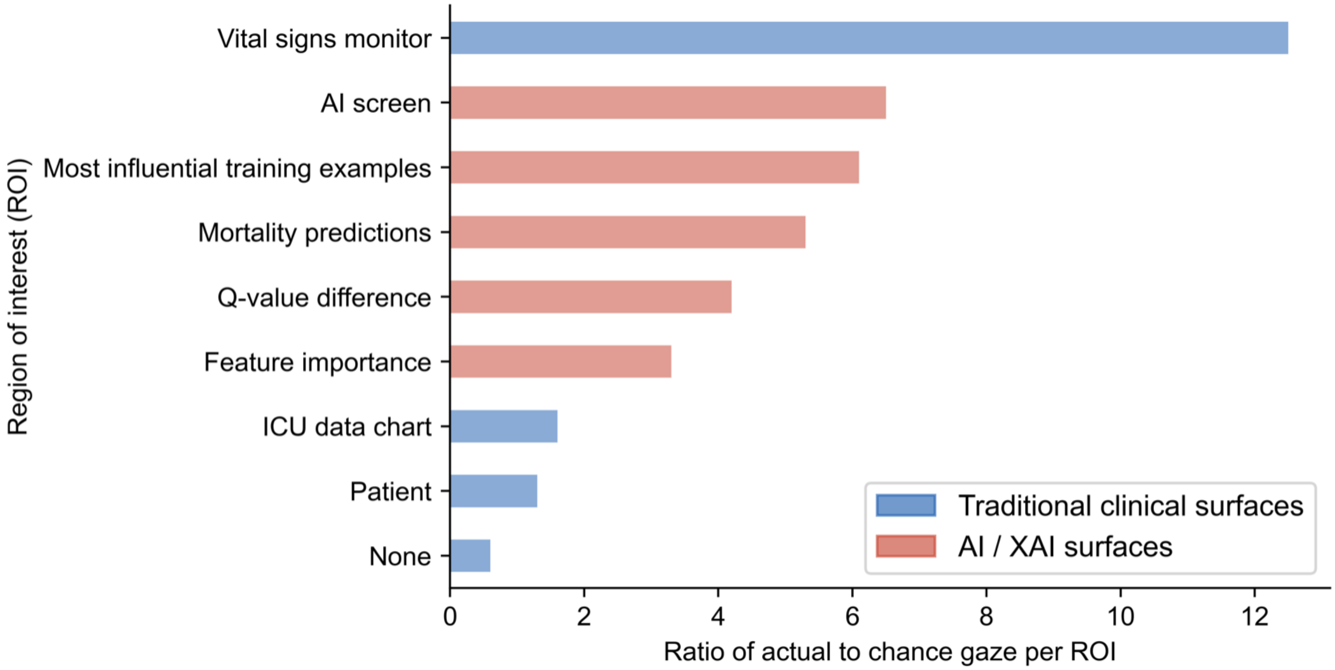

Ratio of actual to chance gaze per ROI. Chance gaze is the proportion expected based on the area occupied by an ROI within the visual field (i.e. if ROI takes up 50% of the screen, 50% of the time, we would expect gaze to randomly fall within it 25% of the time by chance alone). Actual gaze is the proportion observed during the experiment. Both are displayed separately in Supplementary Figure 4, here the ratio of the two is displayed.

SUPPLEMENTARY Figure 6

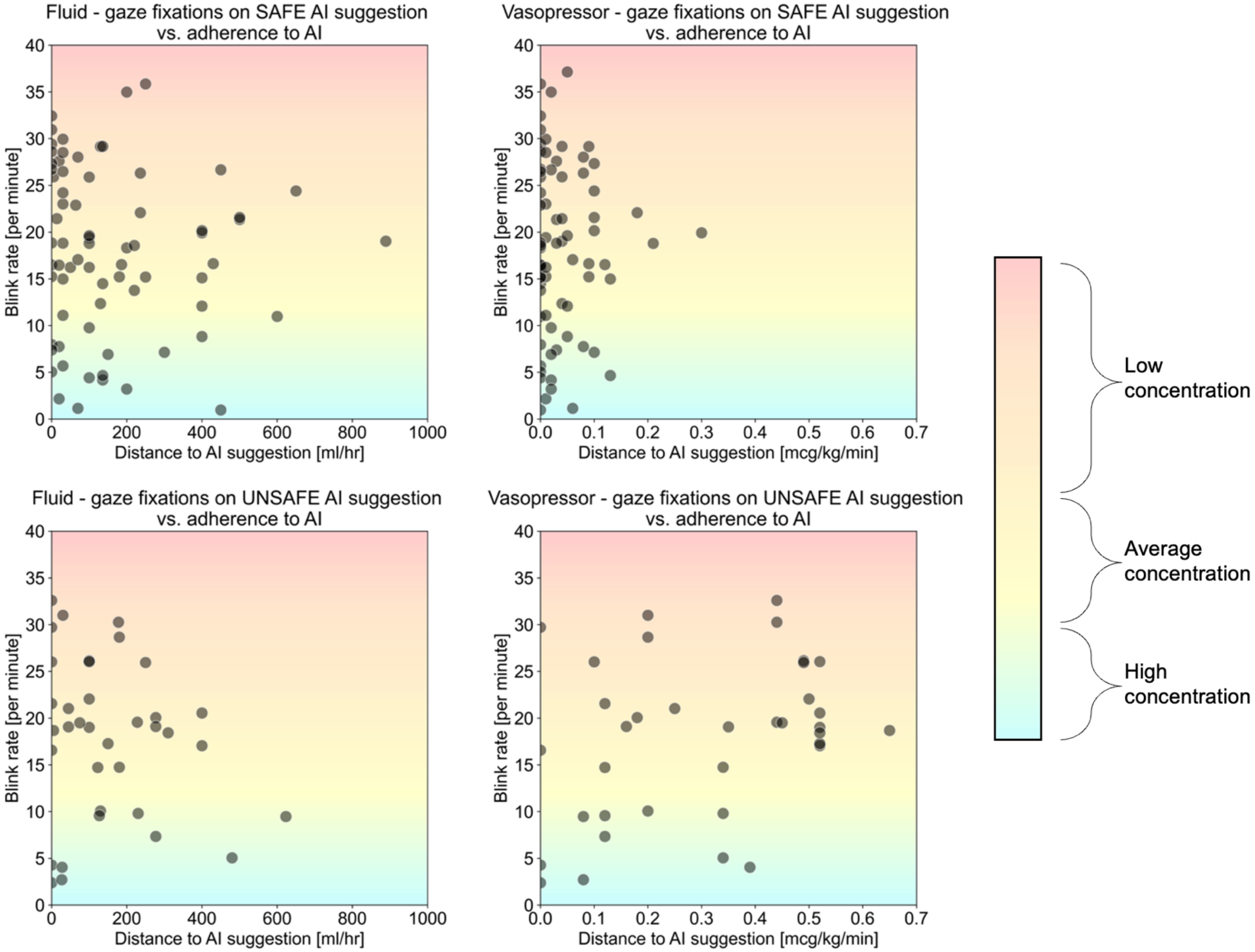

Blink rate by degree of adherence to AI for both fluid and vasopressor and for both safe and unsafe AI suggestions. The degree of adherence to AI is defined by the per trial distance between a doctor's prescription and the AI suggestion. The larger the gap/distance between them, the lower the adherence to AI and vice versa. The background shading of the plots gives an indicator for expected blink rate depending on level of focus/concentration. Typical spontaneous blink rate is around 8-15 with lower levels suggesting higher focus/concentration and vice versa. **Correlation measures:** (safe fluid: r=0.003, p=0.981), (safe vasopressor: r=-0.000, p=0.998), (unsafe fluid: r=-0.076, p=0.649), (unsafe vasopressor: r=0.335, p=0.040).

SUPPLEMENTARY Figure 7

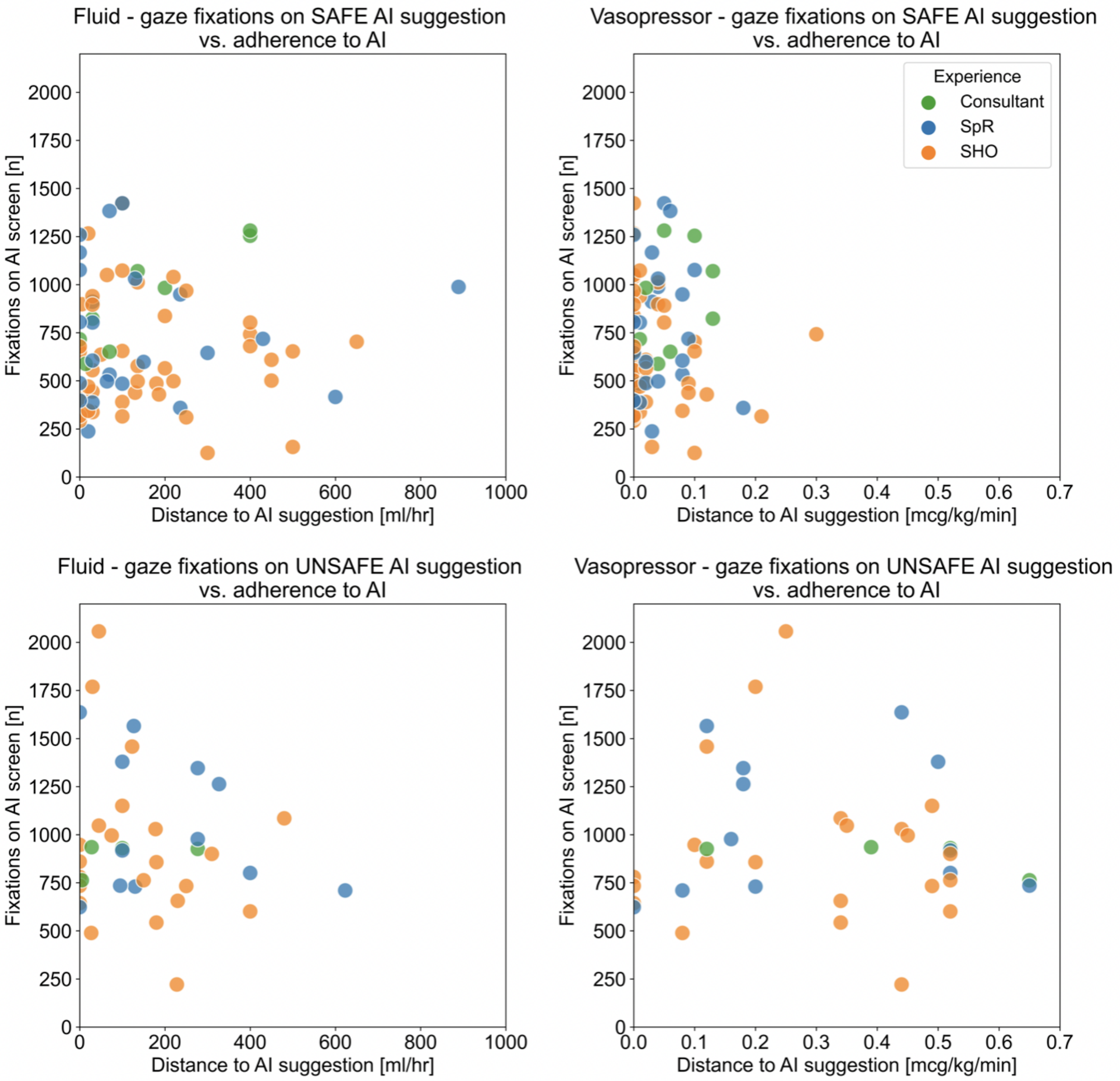

Number of gaze fixations by degree of adherence to AI for both fluid and vasopressor and for both safe and unsafe AI suggestions. The degree of adherence to AI is defined by the per trial distance between a doctor's prescription and the AI suggestion. The larger the gap/distance between them, the lower the adherence to AI and vice versa. Points are also categorised by grade of experience from lowest (SHO) to highest (consultant). **Correlation measures:** (safe fluid: r=0.048, p=0.678), (safe vasopressor: r=-0.021, p=0.856), (unsafe fluid: r=-0.157, p=0.346), (unsafe vasopressor: r=-0.057, p=0.735).

SUPPLEMENTARY Figure 8

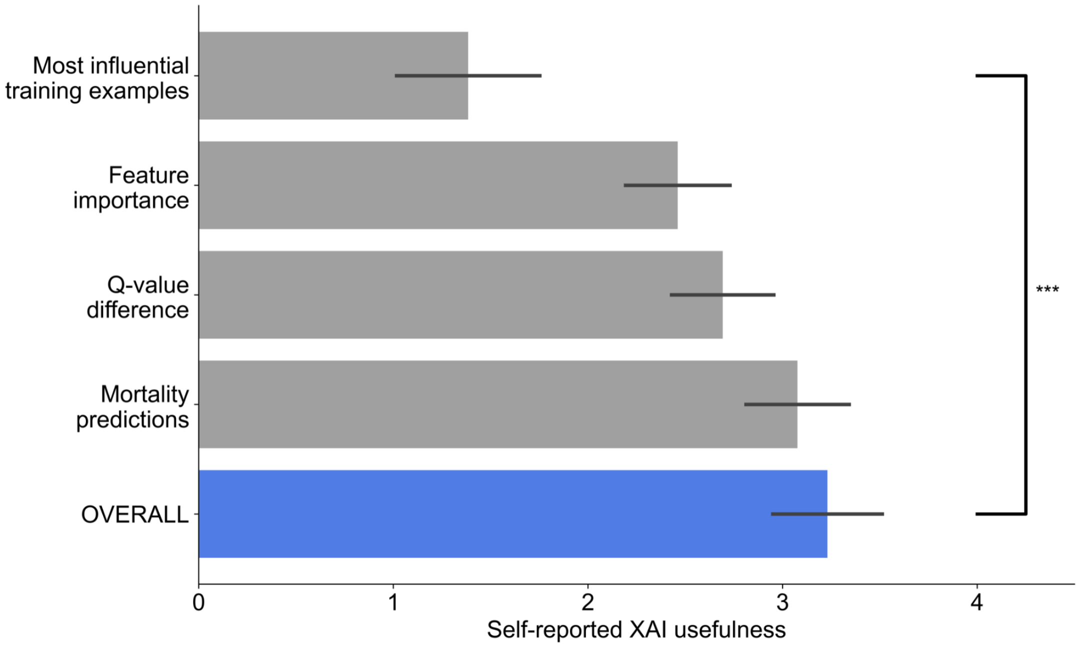

Usefulness rating for each type of explanation as well as overall rating. Mean and SEM error bars. The only significantly different explanation type compared to overall was the 'most influential training examples'. Significance levels are one star for p<0.05 and three stars for p<0.001 (based on Student's t-test).

SUPPLEMENTARY Figure 9

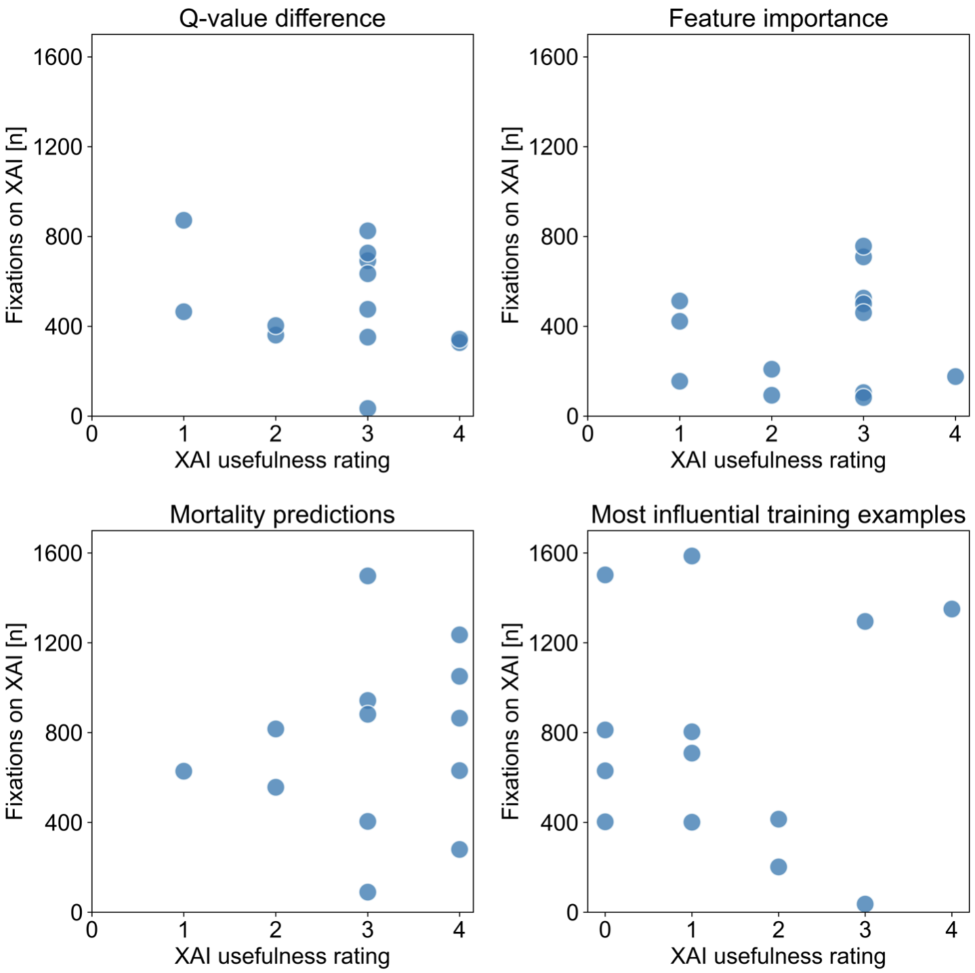

Fixations on XAI vs self-reported usefulness. There was no significant correlation between fixations and usefulness rating for any of the four types of XAI (Q-value difference: r=-0.28, p=0.35, feature importance: r=0.08, p=0.79, mortality predictions: r=0.15, p=0.62, most influential training examples: r=0.01, p=0.97)

Supplementary Note 1: Trial matrix

	Trial					
Subject	1	2	3	4	5	6
3	S	S	U	S	S	U
4	S	U	S	S	S	U
9	S	S	S	U	S	U
10	S	S	S	U	U	S
11	S	S	U	U	S	S
12	S	U	S	U	S	S
13	S	S	U	S	S	U
14	S	S	U	S	U	S
16	S	U	U	S	S	S
17	S	U	S	S	S	U
19	S	U	S	U	S	S
20	S	U	U	S	S	S
21	S	S	S	S	U	U
22	S	S	S	U	S	U
23	S	S	U	S	S	U
24	S	U	S	S	S	U
31	S	S	U	U	S	S
34	S	S	U	S	U	S
36	S	U	U	S	S	S

Subject numbers are non-contiguous in places because the subjects in this experiment (n=19) were a subset of a wider simulation experiment (n=38) for whom eye-tracking data was physically available and of sufficient quality to contribute to the analyses in this manuscript.

Supplementary Note 2: AI explanations

Q-value difference:

This approach leverages the fact that once training is complete, the reinforcement learning (RL) Q table will contain Q values for any given state-action pair. The optimal action is the one with the highest Q value for any given state. However, the difference between this highest Q value and the alternatives might be very small or large. If large, then there is much higher anticipated value from following the recommended action compared to an alternative. This is dichotomised arbitrarily from a continuous value to make interpretation more simple for non-AI users.

Mortality predictions:

This approach leverages the fact that mortality can be predicted for any given state in the RL state space. Therefore the impact of different dosing strategies that might result in transition to alternative states with different predicted mortalities can be displayed to the subject to highlight how alternate strategies might change the risk of death.

Feature importance:

This approach leverages the fact that the state space for RL based sepsis algorithms is commonly constructed using a k-means clustering algorithm to enable dimensionality reduction. After the algorithm converges, the cluster centroids represent the average feature values for patients in a particular state/cluster. A new patient would be assigned to the state/cluster that minimised the distance from their feature values to the respective cluster centroid. Intuitively, with often over 40 features, some features will be closer to the cluster centroid value than others for any patient assigned to a given state. This is exploited to rank features in terms of their proximity to the cluster centroid (or average state feature values) given that the archetypal patient for whom an RL agent policy action most applies is a patient who is most typical of that state. So subjects can be shown the top five ranked features contributing to state assignment.

Most influential training examples:

This approach leverages the fact that the difference in Q values for any given state-action pair between one iteration of Q-learning and the previous iteration reflects how valuable the currently seen training episode is for learning the optimal action for any given state. This is similar to an instance-based explanation used in deep learning imaging XAI where the explanation consists of showing similar image instances from the training instance to explain why a particular image classification has been made.

- Create empty Q and 'Q-difference' tables (both indexed by Q(S,A) tuple)
- For 500,000 episodes:
  - Select random training episode:
    - For each time-step:
      - Perform Q learning
      - Check the Q differences table -> is the Q difference (i.e. difference between old and updated new Q values) from this step among the top 3 for this state-action tuple?
        - If so -> update differences table with the ID for this episode
- End with a dictionary of top 3 influential episodes per Q(S,A) tuple

Supplementary Note 3: Standardised experiment briefing

This simulation experiment aims at studying how clinicians might interact with an AI decision support tool for patients with sepsis. The AI takes patient variables as input and outputs a treatment recommendation for both fluid and vasopressor. We have some evidence of effectiveness of the AI you will interact with on retrospective data from an American dataset and further validation on retrospective data from the Netherlands. However, there has not yet been prospective evidence of either effectiveness or safety.

You will conduct a brief ward round review of 6 ICU patients with sepsis. Between each patient, you will exit the room and be called in again to see the next patient by the nurse.

For each patient, you will first be asked:

- Treatment prescriptions for:
- Fluid in ml for the next hour
- Vasopressor in mcg/kg/min for the next hour
- Your confidence in the prescription on a scale from 1 (low) to 10 (high)
- Whether or not you want to get advice from another doctor / senior doctor

You will then be shown the AI treatment recommendation on a digital screen. The screen contains the AI dose suggestions in the middle (for fluid and vasopressor) along with 4 explanations for the suggestions (one in each corner)

You will then be asked:

- To what extent you agree with the AI suggestion on a scale from 1 (strongly disagree) to 5 (strongly agree)
- Whether you wish to adjust your treatment prescription for:
    - Fluid in ml for the next hour
    - Vasopressor in mcg/kg/min for the next hour
- Whether your confidence in your prescription (on a scale from 1 [low] to 10 [high]) has changed as a result of seeing the AI suggestion
- Whether or not you want to get advice from another doctor / senior doctor
- If the AI suggested treatment was to be administered to the patient, would you act to stop the administration?

We will now show you an example of the AI screen that you will encounter in the experiment. You can see that there is an AI suggestion in the centre of the screen which is how much fluid and vasopressor the AI recommends over the next hour.

Around the corners of the screen there are four different types of explanation which can be thought of as the AI trying to convey the rationale for its suggested doses. The mortality change explanation conveys information on what the AI predicts will happen to the overall mortality risk in the short-term based on potential dose increases or decreases. The most influential training examples explanation conveys which three training cases were most helpful for learning the current suggestion in the same way that we might base our own treatment choices on previous notable cases we learnt from. The feature importance explanation conveys which were the top five features (or items in the data) that were most useful to the AI in generating its current suggestion. The ‘AI treatment options gap’ explanation conveys how much one treatment strategy looks superior compared to alternatives. If all potential options are similar (i.e. a low gap) then it suggests that the AI has near equipoise for options and you may wish to use your own judgement more strongly (as you will have additional information from examining the patient for example). However, if the gap is high it suggests that the AI has identified one particular treatment strategy as superior to the alternatives and therefore it would be worth considering this recommendation more strongly than with a low gap recommendation.

Supplementary Note 4: Pre- and post-experiment questionnaires

Pre-experiment:

- How old are you?
- Gender?
- For how many years have you been working in ICU?
- Are you personally involved, or have experience, in AI research?
- Your opinions on Artificial Intelligence (AI) on a 5-point Likert scale ('Strongly disagree', 'Disagree', 'Neutral', 'Agree', 'Strongly agree')
    - AI will benefit society at large
    - AI will personally benefit me in my day to day life
    - AI will benefit the National Health Service (NHS)
    - AI will personally benefit my work as a clinician
    - I would be comfortable using a validated AI in areas of high clinical uncertainty, such as sepsis resuscitation
    - If we had strong evidence that a doctor assisted by AI was better than a doctor alone at treating sepsis, this AI should be used always and everywhere
    - Widespread use of AI for clinical decision making will lead to deskilling of human physicians
    - If physicians put too much trust in AI, they won't be able to detect when the AI fails, and it will lead to patient harm

The attitude items (the 4 questions on AI benefit) were taken from a scale that we have used in-house for a previously published experiment (Nagendran et al. Quantifying the impact of AI recommendations with explanations on prescription decision making. npj Digit. Med. 2023; 6, 206). The Cronbach's alpha was 0.851 (a measure of internal consistency).

Post-experiment:

- How useful overall were the explanations?
    - 5-point Likert scale ('Not at all useful', 'Not useful', 'Neutral', 'Useful', 'Very useful')

- Please rate the explanation types in usefulness:
    - 5-point Likert scale ('Not at all useful', 'Not useful', 'Neutral', 'Useful', 'Very useful')
    - Repeated separately for each of the four explanations
        - Mortality change
        - Most influential training examples
        - AI treatment options gap
        - Top 5 features

Supplementary Note 5: Patient scenarios and rationale

Patient 1 handover note for participants:

- 50M admitted 2hrs ago from ED with SOB.
- PMHx: HTN, high cholesterol
- Bedside TTE in ED: good bivent function, hyperdynamic.
- CXR: left basal consolidation. COVID -ve.
- ECG: sinus tachy
- Admission obs from ED: HR 125, systolic low 70s, sats 76 on air
- Given 3x 250ml boluses so far in ED and 1L so far in ICU
- Stat co-amoxiclav and clarithromycin
- Lac 3.7 in ED, UO 25ml over last 4 hrs

Mannequin settings:

- Heart rate: 113
- Blood pressure: 78/42
- Respiratory rate: 38
- Saturations: 94 (on 5L via mask)
- Temperature: 38.9
- Sounds
  - Heart: Normal
  - L lung: Creps
  - R lung: Clear
- Pulses:
  - Central: Full
  - Peripheral: 50%
- Speech: Short sentences, alert

AI actions:

- AI safe action
  - Fluid: 900 ml/hr
  - Vasopressor: 0 mcg/kg/min

- AI unsafe action
  - Fluid: 40 ml/hr
  - Vasopressor: 0 mcg/kg/min

Justification:

Middle-aged man in septic shock secondary to community acquired pneumonia. Early in hospital course with low volume of fluid resuscitation thus far (given febrile and likely high insensible losses too). Oliguric and tachypneoic. Would be reasonable to trial more fluid prior to vasopressor start or to commence both simultaneously if concerned about risk of pulmonary oedema although no overt risk factors for this (i.e. no background history of poor cardiac function). Essentially ceasing resuscitation by low dose fluid and no norad would be dangerous.

Patient 2 handover note for participants:

- 84F admitted last night from ED with dysuria, presumed urosepsis. COVID -ve.
- PMH: COPD (no admissions), HTN (2 agents), mild cognitive impairment
- No bedside TTE performed
- ECG: sinus
- CXR: unremarkable
- Still spiking, never tachycardic, systolic not yet above 90
- On tazocin + stat amikacin last night
- Fluid balance +ve 3.5L since admission
- Latest lac 0.7, UO 10-15 ml/hr last 4 hrs

Mannequin settings:

- Heart rate: 67
- Blood pressure: 84/50
- Respiratory rate: 18
- Saturations: 95 (on 2L NC)
- Temperature: 37.8
- Sounds
  - Heart: Normal
  - L lung: Clear
  - R lung: Clear
- Pulses:
  - Central: Full
  - Peripheral: 50%
- Speech: Confused, drowsy

AI actions:

- AI safe action
  - Fluid: 70 ml/hr
  - Vasopressor: 0.09 mcg/kg/min

- AI unsafe action
  - Fluid: 5 ml/hr
  - Vasopressor: 0.75 mcg/kg/min

Justification:

Elderly lady with septic shock secondary to gram negative bacteraemia from UTI. Normally hypertensive and oliguric. Yet to respond to reasonable volume of fluid resuscitation. Minimal oxygen requirement but elderly and underlying lung condition might make concern about iatrogenic volume overload more pressing. Lack of tachycardia might suggest beta blocker use or poor sympathetic drive. Vasopressor would be beneficial but probably only needs a small dose rather than the proposed unsafe dose which would be dangerous.

Patient 3 handover note for participants:

- 42F admitted 8d ago from ED with SOB. COVID +ve pneumonia.
- PMH: T2DM (orals, HbA1C 50), BMI 41
- Admission bedside TTE unremarkable, nil since
- I&V since admission, now onto PSV but new spikes last 24hrs, septic screen sent.
- PSV 10/6 with sats 93 on FiO2 0.45.
- Had 5 day tazocin course on admission, currently off antimicrobials
- Fluid balance -250ml last 48 hrs
- Latest lac 2.3, UO 60-70 ml/hr last 4 hrs

Mannequin settings:

- Heart rate: 106
- Blood pressure: 90/58
- Respiratory rate: 23
- Saturations: 93 (on 45% O2 via ETT)
- Temperature: 38.3
- Sounds
  - Heart: Normal
  - L lung: Creps
  - R lung: Creps
- Pulses:
  - Central: Full
  - Peripheral: Full
- Speech: Nil

AI actions:

- AI safe action
  - Fluid: 50 ml/hr
  - Vasopressor: 0.04 mcg/kg/min

- AI unsafe action
  - Fluid: 100 ml/hr
  - Vasopressor: 0.54 mcg/kg/min

Justification:

Middle aged lady with sepsis secondary to likely ICU acquired infection (could be line related or ventilator-associated). Has been in ICU for over a week so likely to be fluid replete. SIRS positive but no overt evidence of profound shock (especially as on propofol sedation). Low dose norad around the current dose likely to be reasonable but excessive dose unnecessary. Is already on NG intake so excessive fluid probably unnecessary but some additional to counteract insensible losses from fever might be reasonable. High dose norad unnecessary and likely dangerous.

Patient 4 handover note for participants:

- 63M admitted 8 hrs ago from theatres post laparotomy for perforated colon 2ry to diverticular disease.
- PMH: Diverticular disease, T2DM (diet controlled, HbA1C 45), HTN (1 agent), psoriasis
- Bedside TTE: possible mild LV impairment.
- Norad 0.34 (up from peak 0.21 in theatre)
- Fluid balance +ve 6.5L last 12 hrs
- Latest lac 5.8, UO 15ml over last 3 hrs

Mannequin settings:

- Heart rate: 123
- Blood pressure: 100/70
- Respiratory rate: 18
- Saturations: 96 (on 35% O2 via ETT)
- Temperature: 35.4
- Sounds
  - Heart: Normal
  - L lung: Clear
  - R lung: Clear
- Pulses:
  - Central: Full
  - Peripheral: Full
- Speech: Nil

AI actions:

- AI safe action
  - Fluid: 236 ml/hr
  - Vasopressor: 0.38 mcg/kg/min

- AI unsafe action
  - Fluid: 20 ml/hr
  - Vasopressor: 0 mcg/kg/min

Justification:

Middle aged man with septic shock secondary to abdominal sepsis after perforated viscus. Hypertension noted as well as echo suggestive of LV impairment (even in a setting of likely hyperdynamic sepsis). Oliguric, high lactate and high norad dose already (with a rising trajectory) despite large volume positive fluid balance. Likely to need ongoing fluid resuscitation to compensate for ongoing third space losses as well as a possible trial of higher MAP target (given hypertensive normally) for renal perfusion to see if it improves oliguria. Complete cessation of vasopressor would be dangerous.

Patient 5 handover note for participants:

- 33F admitted last night from ED with SOB. COVID -ve.
- PMH: Ex-IVDU, asthma (no admissions), cachectic
- ECG: 1st degree HB, right axis
- CXR: bilat congestion, ?pulmonary oedema vs. infection.
- Bedside TTE: severe AR + MR, possible vegetations.
- Norad 0.04 (up, started 4 hrs ago)
- Fluid balance -250ml last 12 hrs
- Latest lac 4.3, UO 40-50 ml/hr last few hours

Mannequin settings:

- Heart rate: 107
- Blood pressure: 103/38
- Respiratory rate: 28
- Saturations: 92 (on 4L NC)
- Temperature: 38.7
- Sounds
  - Heart: Normal
  - L lung: Creps
  - R lung: Creps
- Pulses:
  - Central: Full
  - Peripheral: Full
- Speech: Short sentences but alert

AI actions:

- AI safe action
  - Fluid: 30 ml/hr
  - Vasopressor: 0.02 mcg/kg/min

- AI unsafe action
  - Fluid: 278 ml/hr
  - Vasopressor: 0.47 mcg/kg/min

Justification:

Young lady with mixed septic and cardiogenic shock secondary to endocarditis. Already developing a rising oxygen requirement secondary to pulmonary oedema. Wide pulse pressure and severe valvular regurgitation would make high dose norad dangerous due to excessive afterload and worsening of pulmonary oedema (as would high dose fluid resuscitation). Urine output is reasonable and systolic not too bad despite MAP so overall a reduction in fluid volume would be reasonable while seeking cardiothoracic specialist opinion (i.e. definitive management).

Patient 6 handover note for participants:

- 29M admitted 8 hrs ago from ED for perineal cellulitis +/- nec fasc.
- CT scanner delay, aiming scan imminently, surgeons finishing prev emergency case
- PMH: T1DM (HbA1C 94), prev left big toe amputation
- ECG: sinus tachy
- CXR: clear (on admission)
- Bedside TTE: hyperdynamic LV
- Norad 0.14, started 3 hrs ago, rising
- Fluid balance +7.5L last 12 hrs
- Latest lac 8.3, UO 80-150 ml/hr last few hours

Mannequin settings:

- Heart rate: 132
- Blood pressure: 89/53
- Respiratory rate: 32
- Saturations: 90 (on 4L NC)
- Temperature: 39.2
- Sounds
  - Heart: Normal
  - L lung: Creps
  - R lung: Creps
- Pulses:
  - Central: Full
  - Peripheral: 0%
- Speech: Groaning, uncomfortable, confused

AI actions:

- AI safe action
  - Fluid: 0 ml/hr
  - Vasopressor: 0.19 mcg/kg/min

- AI unsafe action
  - Fluid: 377 ml/hr
  - Vasopressor: 0.02 mcg/kg/min

Justification:

Young man with septic shock secondary to necrotising fasciitis. Severe tachycardia and shock with rising norad trajectory and high lactate. Urine output is good though. Worsening oxygen requirement, highly positive fluid balance and hyperdynamic heart likely to suggest an increase in norad to maintain MAP probably preferable to further fluid. Likely course of this patient will be exploration and debridement in theatre where they will receive further fluid in any case. Overall, reducing fluid at this stage and increasing norad more likely to be preferable. Sudden drop in norad to 0.02 likely to be dangerous.

Supplementary Note 6: Scenario pre-testing

Prior to the experiment, the six simulated cases (designed by the three doctors in the investigator team) were piloted on 7 doctors from the Critical Care Research Group of Imperial College London.

Each doctor reviewed a subset of 4 of the 6 cases. For each case, there was either a safe or unsafe AI suggestion. Doctors were asked to rate whether they would intervene to stop the treatment suggestion if it were to be automatically administered.

Agreement was calculated by the total proportion of doctors that would correctly stop an unsafe AI suggestion and correctly not stop a safe AI suggestion. Seven doctors x four cases each x two treatment suggestions = 56 data points of which 52 were 'correct' = 93%.

Therefore, overall results of the piloting showed 93% agreement with safe/unsafe ratings.
