# Supplementary material for: Eye tracking insights into physician behaviour with safe and unsafe explainable AI recommendations
Source: NPJ Digit Med. 2024 Aug 2;7:202. doi: 10.1038/s41746-024-01200-x (PMC11297294; doi:10.1038/s41746-024-01200-x)
Supplement: Supplementary file 2 — Reporting Summary [file 41746_2024_1200_MOESM2_ESM.pdf]

nature portfolio

Corresponding author(s): Aldo Faisal

Last updated by author(s): Jul 12, 2024

Reporting Summary

Nature Portfolio wishes to improve the reproducibility of the work that we publish. This form provides structure for consistency and transparency in reporting. For further information on Nature Portfolio policies, see our Editorial Policies and the Editorial Policy Checklist.

Statistics

For all statistical analyses, confirm that the following items are present in the figure legend, table legend, main text, or Methods section.

n/a	Confirmed	
☐	☒	The exact sample size (n) for each experimental group/condition, given as a discrete number and unit of measurement
☐	☒	A statement on whether measurements were taken from distinct samples or whether the same sample was measured repeatedly
☐	☒	The statistical test(s) used AND whether they are one- or two-sided<br>*Only common tests should be described solely by name; describe more complex techniques in the Methods section.*
☒	☐	A description of all covariates tested
☐	☒	A description of any assumptions or corrections, such as tests of normality and adjustment for multiple comparisons
☐	☒	A full description of the statistical parameters including central tendency (e.g. means) or other basic estimates (e.g. regression coefficient) AND variation (e.g. standard deviation) or associated estimates of uncertainty (e.g. confidence intervals)
☒	☐	For null hypothesis testing, the test statistic (e.g. F, t, r) with confidence intervals, effect sizes, degrees of freedom and P value noted<br>*Give P values as exact values whenever suitable.*
☒	☐	For Bayesian analysis, information on the choice of priors and Markov chain Monte Carlo settings
☒	☐	For hierarchical and complex designs, identification of the appropriate level for tests and full reporting of outcomes
☒	☐	Estimates of effect sizes (e.g. Cohen's d, Pearson's r), indicating how they were calculated

Our web collection on statistics for biologists contains articles on many of the points above.

Software and code

Policy information about availability of computer code

Data collection	Google sheets, Pupil Core headset, Pupil Labs software (Core, version 3.3)
Data analysis	Python 3.9.0 with following packages: matplotlib, numpy, pandas, pickleshare, scipy, scikit-learn, seaborn. Full details available in the readme at https://doi.org/10.6084/m9.figshare.23192615

For manuscripts utilizing custom algorithms or software that are central to the research but not yet described in published literature, software must be made available to editors and reviewers. We strongly encourage code deposition in a community repository (e.g. GitHub). See the Nature Portfolio guidelines for submitting code & software for further information.

Data

Policy information about availability of data

All manuscripts must include a data availability statement. This statement should provide the following information, where applicable:

- Accession codes, unique identifiers, or web links for publicly available datasets
- A description of any restrictions on data availability
- For clinical datasets or third party data, please ensure that the statement adheres to our policy

The data (in CSV format) and code that support the findings of this study are available online at: https://doi.org/10.6084/m9.figshare.23192615

Research involving human participants, their data, or biological material

Policy information about studies with human participants or human data. See also policy information about sex, gender (identity/presentation), and sexual orientation and race, ethnicity and racism.

Reporting on sex and gender	N/A
Reporting on race, ethnicity, or other socially relevant groupings	N/A
Population characteristics	ICU doctors in London
Recruitment	ICU physicians were recruited as participants using both targeted advertising to a local NHS trust (Imperial College Healthcare NHS Trust) and convenience sampling with the following inclusion criteria: (i) practising physician, (ii) has worked for at least 2 months in an adult ICU, (iii) currently works in ICU or has worked in ICU within the last 6 months. Each experiment lasted approximately 60 minutes in total and participants were compensated for their time. All participants provided informed consent to participate.
Ethics oversight	The study was approved by the Research Governance and Integrity Team (RGIT) at Imperial College London and the Health Research Authority (Ref: 22/HRA/1610).

Note that full information on the approval of the study protocol must also be provided in the manuscript.

Field-specific reporting

Please select the one below that is the best fit for your research. If you are not sure, read the appropriate sections before making your selection.

☐ Life sciences ☒ Behavioural & social sciences ☐ Ecological, evolutionary & environmental sciences

For a reference copy of the document with all sections, see nature.com/documents/nr-reporting-summary-flat.pdf

Behavioural & social sciences study design

All studies must disclose on these points even when the disclosure is negative.

Study description	High fidelity human-AI interaction study using eye-tracking of ICU doctors in a simulation suite
Research sample	London ICU doctors
Sampling strategy	Convenience sampling with targeted advertising
Data collection	Google sheets, Pupil Core headset, Pupil Labs software (Core, version 3.3)
Timing	27 Jul 2022 - 28 Oct 2022
Data exclusions	No data exclusions per se -> although this experiment was a sub-study of a larger simulation suite study, see https://www.medrxiv.org/content/10.1101/2023.10.03.23296437v1. The sub-study forming this manuscript included data only for the 19 of 38 participants in the simulation suite with eye-tracking data available (as this sub-study was focused specifically on eye-tracking).
Non-participation	See above on data exclusions
Randomization	No randomization

Reporting for specific materials, systems and methods

We require information from authors about some types of materials, experimental systems and methods used in many studies. Here, indicate whether each material, system or method listed is relevant to your study. If you are not sure if a list item applies to your research, read the appropriate section before selecting a response.

Materials & experimental systems

n/a	Involved in the study
☒	☐ Antibodies
☒	☐ Eukaryotic cell lines
☒	☐ Palaeontology and archaeology
☒	☐ Animals and other organisms
☒	☐ Clinical data
☒	☐ Dual use research of concern
☒	☐ Plants

Methods

n/a	Involved in the study
☒	☐ ChIP-seq
☒	☐ Flow cytometry
☒	☐ MRI-based neuroimaging
